# *Ex Vivo* and *In Silico* Feasibility Study of Monitoring Electric Field Distribution in Tissue during Electroporation Based Treatments

**DOI:** 10.1371/journal.pone.0045737

**Published:** 2012-09-20

**Authors:** Matej Kranjc, Franci Bajd, Igor Sersa, Eung Je Woo, Damijan Miklavcic

**Affiliations:** 1 Faculty of Electrical Engineering, University of Ljubljana, Ljubljana, Slovenia; 2 Institut Jozef Stefan, Ljubljana, Slovenia; 3 Department of Biomedical Engineering, Kyung Hee University, Seoul, Republic of Korea; University of California, Berkeley, Berkeley, United States of America

## Abstract

Magnetic resonance electrical impedance tomography (MREIT) was recently proposed for determining electric field distribution during electroporation in which cell membrane permeability is temporary increased by application of an external high electric field. The method was already successfully applied for reconstruction of electric field distribution in agar phantoms. Before the next step towards *in vivo* experiments is taken, monitoring of electric field distribution during electroporation of *ex vivo* tissue *ex vivo* and feasibility for its use in electroporation based treatments needed to be evaluated. Sequences of high voltage pulses were applied to chicken liver tissue in order to expose it to electric field which was measured by means of MREIT. MREIT was also evaluated for its use in electroporation based treatments by calculating electric field distribution for two regions, the tumor and the tumor-liver region, in a numerical model based on data obtained from clinical study on electrochemotherapy treatment of deep-seated tumors. Electric field distribution inside tissue was successfully measured *ex vivo* using MREIT and significant changes of tissue electrical conductivity were observed in the region of the highest electric field. A good agreement was obtained between the electric field distribution obtained by MREIT and the actual electric field distribution in evaluated regions of a numerical model, suggesting that implementation of MREIT could thus enable efficient detection of areas with insufficient electric field coverage during electroporation based treatments, thus assuring the effectiveness of the treatment.

## Introduction

Electrochemotherapy (ECT) and nonthermal irreversible electroporation ablation (NTIRE) are potent procedures used in solid tumor treatment. In addition, NTIRE is also promising ablation method for nonmalignant tissues. Both, ECT and NTIRE, rely on cell membrane electroporation, a process which increases cell membrane permeability due to externally applied electric field [1]–[3]. ECT combines electroporation with the use of chemotherapeutic drugs, which exhibit higher cytotoxicity when they are combined [4]–[7]. ECT has been successfully used for treatment of cutaneous and subcutaneous metastasis of various cancers achieving over 70% complete responses [6] on over 3000 treated patients in Europe since 2006 [8]. In NTIRE the extensive membrane electroporation alone leads to a loss of cell homeostasis and finally to cell death [9], [10].

Recently, ECT and NTIRE have been also used in treatment of deep-seated tumors [11], [12]. Electroporation based treatments efficiency is correlated to electric field distribution [13]. More specifically, at a given number and duration of pulses, the local electric field is the critical factor determining tissue electroporation. In order to ensure adequate electric field coverage of the treated tissue treatment, planning using numerical modeling was introduced [14], [15]. It also needs to be noted that cell membrane conductivity and consequently cell/tissue conductivity are increased after electroporation in a nonlinear way [16], [17]. Unfortunately, this nonlinear tissue conductivity increase due to electroporation along with uncertainty of tissue conductivity determination makes treatment plan inaccurate and thus inherently unreliable. Another factor influencing success of the electroporation treatment is inaccuracy in electrode positioning with respect to the target tissue [14], [18]. Both, tissue conductivity and electrode positioning were also highlighted as major unknowns in a recent publication on patient specific treatment planning for electroporation based therapies [19]. It is therefore important to find efficient means of electroporation process monitoring on site. Two promising methods for monitoring electroporation are electrical impedance tomography (EIT) and magnetic resonance imaging (MRI). EIT was already successfully applied in vivo [20], [21] however it introduces demanding implementation of additional electrodes and solving the mathematically ill-posed problem of determining conductivity from boundary voltage measurements. MRI was also used to detect electroporated tissue regions, however not *in situ* and only for irreversible electroporation based treatments such as NTIRE [22], [23]. Recently, a method based on magnetic resonance electrical impedance tomography (MREIT) was suggested for monitoring an electric field distribution during irreversible and also reversible electroporation based treatments such as ECT [24]. MREIT would namely allow determination of electric field distribution *in situ* thus taking into account actual tissue conductivity and electrodes position. Since electric field distribution is available immediately after the delivery of electric pulses also corrective intervention would be possible.

The aim of this study was to investigate whether electric field distribution during electroporation of *ex vivo* liver tissue can be efficiently monitored by MREIT. In addition, feasibility of using MREIT to monitor electric field distribution during electroporation based treatments was evaluated *in silico* on a recently reported case [11].

## Materials and Methods

### Preparation of ex vivo Tissue Sample

**Figure 1:**
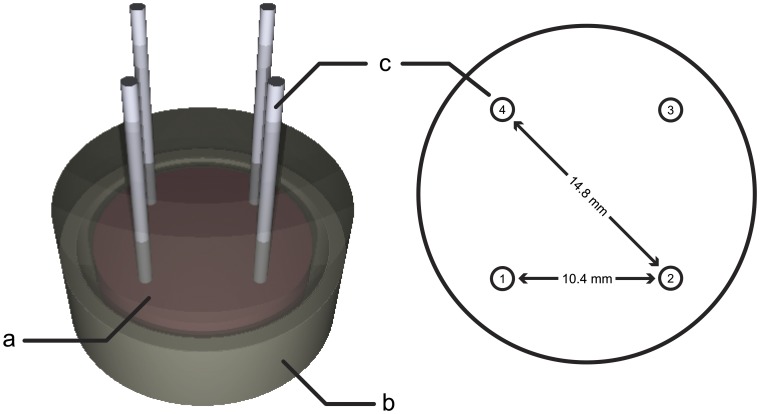
Liver sample with inserted electrodes placed in an acrylic glass container. A liver sample (a) was placed in an acrylic glass container (b). Four needle electrodes (c) were inserted in the sample through predesigned holes in the container. Sequences of electric pulses were delivered between electrode pairs 1–2 and 1–3.

Chicken liver primarily intended for human consumption were obtained from a slaughterhouse (Perutnina Ptuj, d.d., Ptuj, Slovenia) which operates in accordance to Slovenian law. Experiments were in compliance with the slaughterhouse as all of their goods are produced strictly for human consumption. The process of slaughtering is regulated by Rules on animal protection and welfare at slaughter (Ur.l. RS, N. 5/2006) which ensures ethical standards of slaughtering procedure and is in compliance with European Union Council directive on the protection of animals at the time of slaughter or killing (93/119/EC). Temperature of the tissue was maintained at 4°C before the beginning of experiment when they were allowed to heat up to the room temperature. Tissues were sectioned in flat and cylindrical shaped samples with a diameter of 20 mm and placed in an acrylic glass container (see Fig. 1). Four cylindrically shaped platinium-iridium electrodes with a diameter of 1 mm were placed inside. Samples were then inserted in the 25 mm RF probe and connected to the electric pulse generator using cables including a low-pass filter to avoid possible RF disturbances in the NMR signal. The sequence of four high voltage electric pulses with an amplitude *U*_el_ of either 1000 V or 1500 V and a duration of 100 µs were delivered between electrode pairs 1–2 and 1–3 by an electric pulse generator Cliniporator Vitae (IGEA, Carpi, Italy) to establish electric field distribution below and above reversible electroporation threshold value in the case of *U*_el_  = 1000 V and *U*_el_  = 1500 V, respectively. Electroporation threshold values were already determined in previous study [25] and were adjusted to our pulse parameters [26]. The current and voltage of electric pulses were also measured with an oscilloscope (WavePro 7300A, LeCroy, USA) using current probe (AP015, LeCroy, USA) and high-voltage probe (PPE2KV, LeCroy, USA). All experiments were repeated three times for each electrode/voltage arrangement. The sample was replaced with a fresh one after each electroporation pulse delivery to ensure identical initial conditions in all electroporation experiments.

### Magnetic Resonance Electrical Impedance Tomography

MREIT algorithms are in general used for reconstruction of conductivity distribution inside samples [27], [28], although they can also be applied for reconstruction of electric field. MREIT is based on current density imaging (CDI) which is an MRI method for acquiring current density distribution inside samples. Briefly, in CDI, maps of image signal phase shift are acquired after application of electric current pulses to the sample [29]. The phase shift is proportional to the average magnetic field change in the sample (in the direction of the static magnetic field) caused by currents flowing through the sample. By rotating sample along with the electrodes to different perpendicular orientations, different vector components of electric current induced magnetic field change can be obtained from current induced phase shift *φ* stored in acquired images
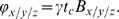
(1)where *γ* is the proton gyromagnetic ratio and *t*_c_ is the total duration of the applied electric pulses. Once these are known, electric current density in the sample **J**  =  (*J*_x_, *J*_y_, *J*_z_) can be calculated from the current induced magnetic field change vector **B** *_c_*  =  (*B*_x_, *B*_y_, *B*_z_) using Ampere’s law



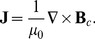
(2) As rotating of the sample to different orientations can cause unwanted pixel misalignments and sample deformation, geometry of *ex vivo* tissue presented in this study was such that currents were flowing predominately in the plane perpendicular to the electrodes. This allowed a simpler current distribution reconstruction by disregarding two negligible in-slice magnetic field change components (*B*_x_, *B*_y_) and using only the nonzero component in direction parallel to the imaging slice, i.e. *B*_z_, for calculation of the current density **J**_CDI_ = (*J*_x_, *J*_y_)
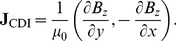
(3)


**Figure 2:**
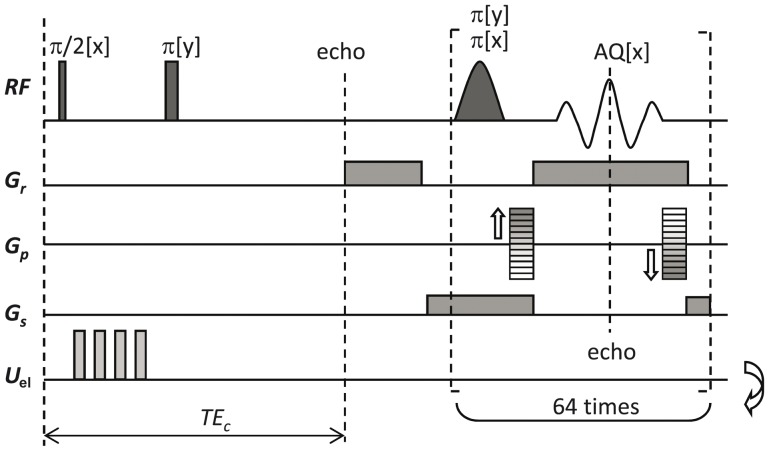
Two-shot RARE CDI sequence.

MR images of current induced magnetic field changes were obtained by means of two-shot RARE CDI sequence [30] as shown in Fig. 2 with parameters: imaging matrix 64×64, field of view 30 mm, inter-echo delay 2.64 mm, echo time of the current encoding period 20 ms and the time interval between two RARE signal acquisitions was 10 s. MR imaging was performed on a TecMag NMR spectrometer connected to an Oxford 2.35 T horizontal bore superconducting magnet. The MRI system was equipped with Bruker microimaging accessories with maximum gradients of 250 mT/m.

After current density inside the sample was obtained, the MREIT J-substitution algorithm was applied. The algorithm is based on iteratively solving of Laplace’s equation:
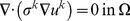
(4)where *u* satisfies the following boundary conditions:
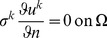
(5)




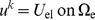
(6)where Ω is the sample body, Ω_e_ is the boundary of the electrodes, *n* denotes normal vector to the boundary, *U*_el_ is the voltage on the electrodes measured by the generator, *σ* is unknown electrical conductivity of the sample and *k* denotes a number of current iteration. Electrical conductivity was updated after each iteration (*k*+1)
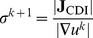
(7)where 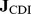 is current density obtained by CDI method. When difference between two successive conductivities *σ* falls below 0.01 electric field distribution E can be calculated using Ohm’s law



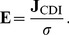
(8)Calculations were performed with the numerical computational environment Matlab 2011a (Mathworks, Natick, MA) on a desktop PC (Windows 7, 2.66 GHz, 4 GB RAM) using a finite element method.

### 3-D Numerical Model

In order to evaluate the proposed MREIT algorithm used to acquire the current and electric field distribution during electroporation based treatments without rotating the subject, i.e. using only one **B** component, we performed a simulation in the case of a 3-D numerical model designed for the purpose of an electrochemotherapy treatment of deep-seated liver tumors. The treatment was done as part of an on-going Phase I/II clinical study (EudraCT number 2008-008290-54; clinicaltrials.org – NCT01264952). The study was approved by Institutional Medical Board and Ethical Committee of the Republic of Slovenia. A report of the treatment was published by Edhemovic *et al*, where all details about the treatment procedure can be found [11].

**Figure 3:**
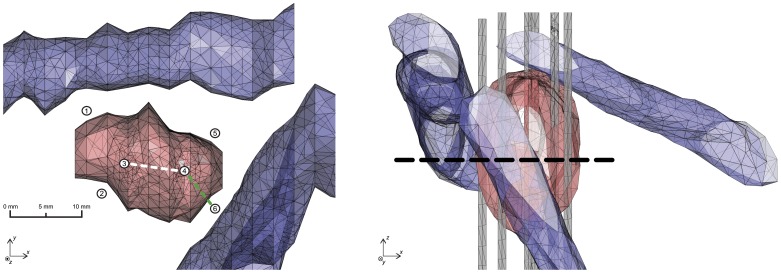
3-D numerical model of a deep-seated tumor in a liver. A 3-D numerical model of a deep-seated tumor in a liver observed from two different viewing angles. The tumor (in red) was located between the IVC and main hepatic veins (all in blue). The liver tissue, surrounding the tumor and veins, is not shown for the purpose of visualization. Electrodes (in grey) are labeled with numbers from 1 to 6. Two regions in an *xy*-plane in the middle of the tumor (black dashed line) were evaluated: the tumor region (between electrodes no. 3 and 4; white dashed line) and the region consisted of a tumor and a liver tissue (between electrodes no. 4 and 6; green dashed line).

Briefly, a model of a patient with a metastasis located between the inferior vena cava (IVC) and the main hepatic veins was studied. The model included a 3-D geometry of the metastasis that was built by means of segmented MRI images of the patient [31]. The numerical model distinguishes between three tissues as shown in Fig. 3: liver, tumor and blood vessels. Six electrodes were inserted in a configuration as obtained after genetic algorithm optimization [32]. For the purpose of *in silico* MREIT evaluation we focused on two regions between two pairs of electrodes where electroporation occurs due to the highest electric field. The first region or the tumor region was situated between the electrode pair 3–4. The tumor had a homogeneous electrical conductivity *σ*_T_ = 0.4 S/m. The second region or the tumor-liver region was situated between the electrode pair 4–6. The region consisted of tumor and liver tissues, each with its own electrical conductivity. The liver had a homogeneous electrical conductivity *σ*_L_ = 0.05 S/m. On the border between tumor and liver tissue we applied gradual change of electrical conductivity using sigmoid curve.

The magnetic field density B*  =  (*B_x_*, *B_y_, B_z_)* and the electric field distribution E*  =  (*E_x_*, *E_y_, E_z_)* were calculated for two regions – the tumor and the tumor-liver region by applying electric pulses with amplitudes of *U*_app_ = 1700 V and *U*_app_ *= *2100 V between each pair of electrodes (3–4 and 4–6, respectively). The obtained electric field distribution E* can be considered as an actual electric field distribution. It was used as a reference in evaluation of MREIT results using only one B* component, i.e. *B*_z_, established during application of pulses in one direction (either between electrodes pair 3–4 or 4–6). These calculations were done by solving the 3-D numerical model using the finite element method with the commercial finite element software package COMSOL Multiphysics 3.5a (COMSOL AB, Stockholm, Sweden). The current density distribution **J**_CDI_ = (*J*_x_, *J*_y_) was calculated using Eq. (3) by means of only the component *B_z_* in an *xy*-plane positioned across the middle of the tumor (see Fig. 3). Electric field distribution 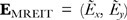) within the *xy*-plane was calculated using Ohm’s law
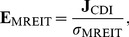
(9)where *σ*_MREIT_ is electrical conductivity distribution of liver and tumor obtained by the J-substitution algorithm as described in [24].

## Results

**Figure 4:**
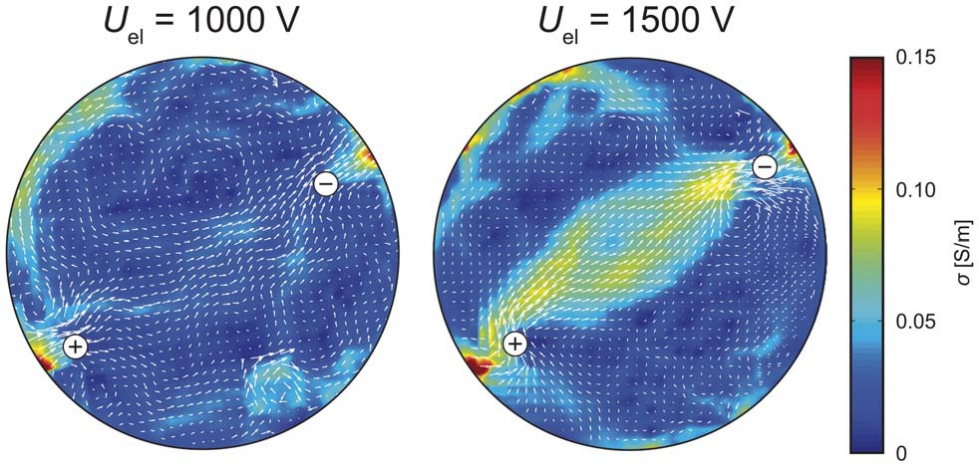
Electrical conductivitiy of a liver tissue obtained by MREIT. An electrical conductivity of a liver tissue obtained by MREIT. Tissues were exposed to four 100 µs long electric pulses with an amplitude of *U*_el_  = 1000 V (left figure) and 1500 V (right figure). Pulses were delivered between two needle electrodes (marked with + and −). The corresponding electric current densities are presented as a vector field (white arrows). A length of arrows corresponds to current density magnitude.

When a tissue sample is exposed to electroporation pulses an electric current density and electric field are established inside the tissue. The former was successfully measured by the CDI method using only the *B*_z_ component, while the later was obtained from the CDI data using the MREIT J-substitution algorithm. Electrical conductivities, calculated using the MREIT algorithm, are presented together with current densities in Fig. 4, which correspond to the experiment on the liver tissues exposed to four 100 µs long electroporation pulses with different amplitudes (*U*_el_ = 1000 V and *U*_el_ = 1500 V). When tissue was exposed to electric pulses with an amplitude of *U*_el_  = 1000 V no significant alteration of electrical conductivity was measured except near boundaries and in the region of inactive electrode. On contrary, major changes of conductivity were observed in the region between the electrodes compared to the rest of the tissue when it was exposed to pulses with *U*_el_  = 1500 V. Current density was at both applied amplitudes the highest near the active electrodes and in the region of increased conductivity in the case of *U*_el_  = 1500 V.

**Figure 5:**
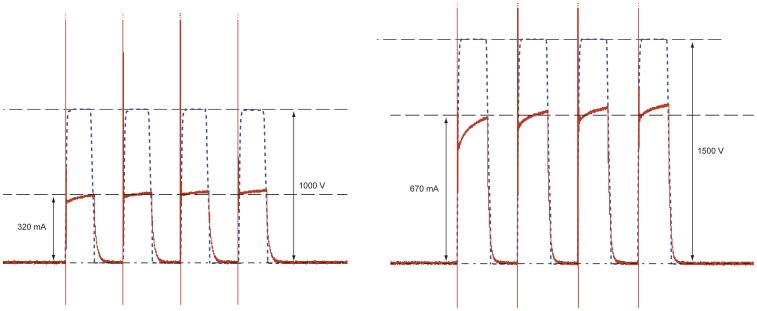
Electric current and voltage in liver tissue. Electric current (red solid line) and voltage (blue dashed line) in liver tissue exposed to four 100 µs long electric pulses with an amplitude of *U*_el_  = 1000 V (left figure) and 1500 V (right figure).

Measured electric current and voltage for liver tissues exposed to four 100 µs long electroporation pulses with different amplitudes (*U*_el_  = 1000 V and *U*_el_  = 1500 V) are presented in Fig. 5.

**Figure 6:**
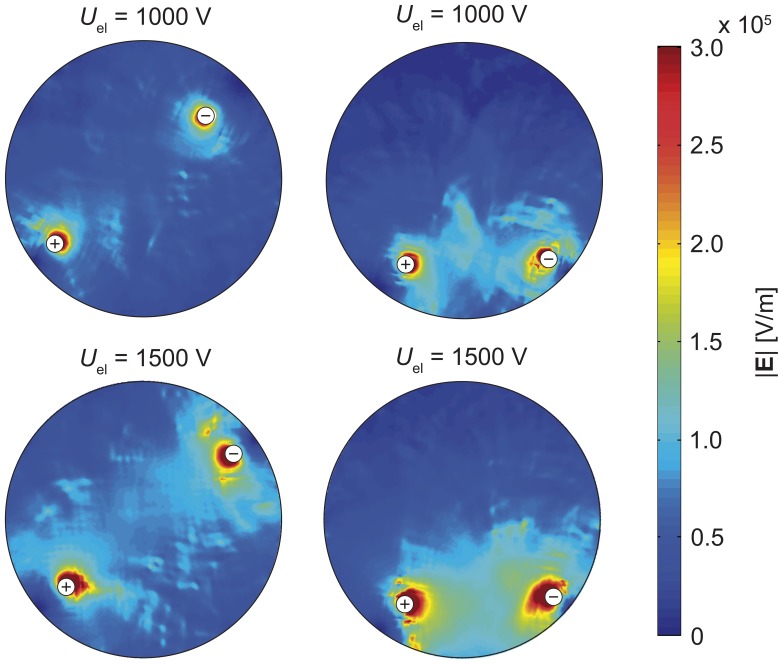
Electric field distribution in the liver tissue obtained by MREIT. Electric field distribution in the liver tissue exposed to the sequence of four electric pulses of different amplitudes (*U*_el_  = 1000, 1500 V). All four distributions are scaled to the same range for easier comparison.

Electric field distributions in the liver tissue for electric pulses with different amplitudes (*U*_el_  = 1000 V and *U*_el_  = 1500 V) delivered between the electrode pairs 1–2 and 1–3 are shown in Fig. 6. As expected, the electric field was the highest around electrodes and was larger in *U*_el_  = 1500 V case than in *U*_el_  = 1000 V, as well as higher in case when pulses were delivered between electrodes pair 1–2 than between 1–3. Results were successfully and reproducibly obtained in all treated liver tissues.

**Figure 7:**
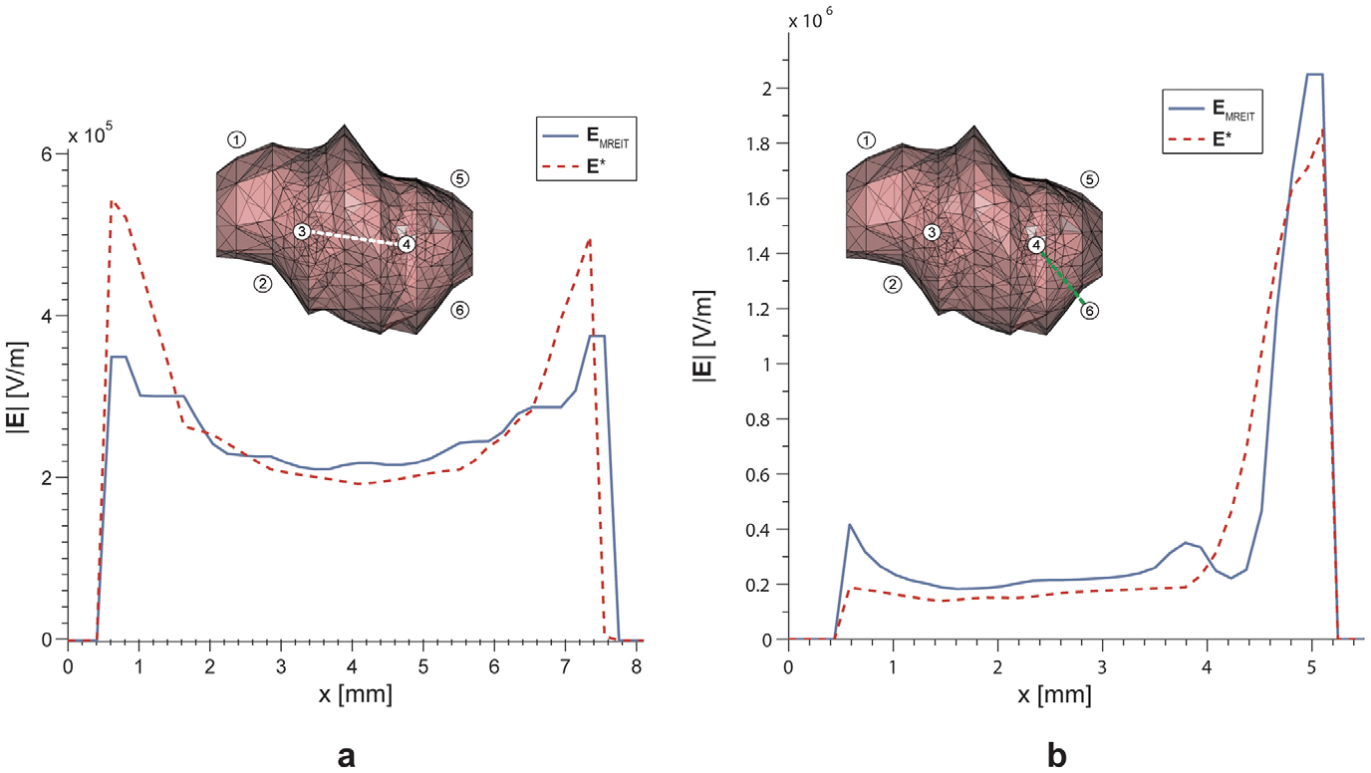
Electric field distribution in the 3-D numerical model obtained by MREIT. An electric field distribution across the tumor region (a) and in the tumor-liver region (b) obtained by means of the MREIT algorithm using only the *B*_z_ component (blue solid line) and the corresponding true electric field calculated by the numerical model (red dashed line). Evaluations regions are presented as white and green dashed line in the inserts of figure. Voltage of 1700 V and 2100 V was applied between the electrode pair 3–4 and 4–6, respectively. Note that scales in a and b are different.

Evaluation results of MREIT to monitor electric field distribution during electroporation based treatments are presented in Fig. 7. An electric field distribution across the tumor region and in the tumor-liver region obtained by means of the MREIT algorithm using only the *B*_z_ component is compared with the corresponding true electric field calculated by the 3-D numerical model of a deep-seated tumor in liver.

## Discussion

The aim of this study was to investigate feasibility of the MREIT technique to monitor electric field distribution during tissue electroporation by means of an *ex vivo* liver tissue experiment. Feasibility of MREIT to use in electroporation based treatments such as electrochemotherapy and nonthermal irreversible electroporation ablation was evaluated also using numerical modeling on a recently reported case.

As shown in Figs. 4 and 6, electric field and electric conductivity distributions within liver tissue during application of electroporation pulses were successfully obtained. When examining results and comparing them to our previous findings on agar phantoms, considerable differences were however observed [24]. While dielectric properties of agar remained unchanged in spite of applied high electric field, presumably due absence of a cell structure in an agar gel [33], significant changes of the liver tissue electrical conductivity were observed when tissue was exposed to pulses with an amplitude of *U*_el_ = 1500 V (Fig. 4). The region with a higher electrical conductivity between electrodes was established as a consequence of tissue changes associated to a high electric field in the region. Such changes were not detected in tissue exposed to pulses with an amplitude of *U*_el_ = 1000 V due to lower electric field. Observed tissue changes are a consequence of local tissue electroporation as electric field in the region between electrodes exceeded reversible threshold value in the case of *U*_el_ = 1500 V whereas most of this region remained under the threshold value in the case of *U*_el_ = 1000 V. Comparison of electric current (Fig. 5) also confirms increment of electrical conductivity as amplitudes value of electric current in liver tissue approximately doubled (from 320 mA to 670 mA) when the voltage was increased for only half of its value (from 1000 V to 1500 V). Similar changes of tissue conductivity after electroporation were already observed and have been reported previously [17], [34]. It also needs to be noted that local alterations of conductivity near the boundary of the container and in the region of inactive electrode in Fig. 4 are measurement errors due to distortions of magnetic field caused by electrodes and due to noise near the boundary where the lower limit of sensitivity in CDI is met.

Implementation of MREIT during electroporation pulse delivery could significantly improve electroporation procedures in clinical applications such as ECT and NTIRE. Adequate electric field distribution and sufficiently high local electric field are two of the most important conditions for successful realization of both therapies. Even though the treatment planning for ECT already proved in clinical treatment of deep-seated tumors [14], its applicability is limited for now due to conductivity values of treated tissues which were determined with high uncertainty. As a consequence treatment plan can yield inappropriate electric pulse parameters and electrode configuration. Another concern is electrodes positioning during the treatment procedure as it is difficult to place electrodes accurately according to the treatment plan [18]. An imprecise insertion of the electrodes can establish inadequate electric field coverage of the treated tissue which can lead to a treatment failure. Monitoring of the electric field distribution during ECT and NTIRE by means of MREIT would thus enable detection of insufficient electric field coverage before potential treatment failure, hence assuring and increasing the effectiveness of both methods. Some previously reported difficulties associated with the use of MREIT [24] have been addressed as part of this study. We have successfully evaluated a major concern of the MREIT use, namely, whether it is possible to obtain an accurate electric field distribution from only one **B** component using a 3-D numerical model built for the purpose of electrochemotherapy treatment of deep-seated tumors in liver. An electric field was successfully obtained in both regions, i.e. in the tumor region and in the tumor-liver region. As shown in Fig. 7a and 7b, a good agreement was obtained in both regions. It should be noted that regions with tissues of different conductivities correspond in MREIT to only one current density distribution due to non-uniqueness [35]. This necessitates application of more than one sequence of pulses in at least two directions in order to calculate conductivity and electric field distributions more accurately. This approach is usually done in regular MREIT for diagnostic cases where injected currents are of the order of few mA and dielectric properties of the observed tissue remain intact [36]. However, this cannot be done in treatment cases, such as electroporation, where high electric currents affect electrical conductivity of the tissue after each pulse sequence [17], [34]. Even though, results obtained by means of using only one pulse sequence seem sufficiently accurate to enable monitoring of electric field and consequently assume tissue electroporation in the target tissue.

In this study new potential in monitoring electric field distribution by means of MREIT were examined. Electric field distribution in tissue samples *ex vivo* was measured during electroporation by applying electrical pulses in one direction only. As expected, alteration of tissue conductivity distribution caused by applied high voltage pulses was detected. Conductivity changes that occur during the pulse [21] are at the moment too demanding to assess with MREIT as a function of time. Even though, it is important to be aware that with the CDI technique the accumulative effect of electric current on the MRI signal phase is measured. Therefore, this technique yields a current density distribution, which is a time average of its altering time course so that all the consequences of conductivity alteration, which affect electric current, are not neglected within this distribution. It was also shown that it is possible to obtain a corresponding electric field distribution in electroporation based treatments using numerical model based on a clinical case of the ECT treatment. Our results show that MREIT would confirm delivery of sufficiently high electric field in the whole target tissue and thus enable detection of areas with insufficient electric field coverage during electroporation based treatments like electrochemotherapy and nonthermal irreversible electroporation ablation. This essential near real time information could then be used to improve the electroporation treatment by setting new amplitudes of electric pulses, changing their duration or number or by electrode repositioning, thus increasing effectiveness of electroporation based clinical procedures.
